# Supplementary material for: Content-rich biological network constructed by mining PubMed abstracts
Source: BMC Bioinformatics. 2004 Oct 8;5:147. doi: 10.1186/1471-2105-5-147 (PMC528731; doi:10.1186/1471-2105-5-147)
Supplement: Additional File 2 — The original results of the above study (non-essential files are deleted to keep the file size under the limit set by BMC bioinformatics). [file 1471-2105-5-147-S2.bz2 › chilibotAdditionalFile2/dip05/197ID8652557E680/html/ERCC3_GTF2H1.html]

 


 **ERCC3** and **GTF2H1** 
  
Found 427 abstracts in PubMed, retrieved 05.  
 

 What does Google say? 
 PDF only 
| .edu only 

---

**Interactive relationship** (e.g. stimulation, inhibition, etc)

**Neutral relationship**- Moreover, when XPD mutations prevent interaction with the p44 subunit of TFIIH  [ **GTF2H1** ]   [ **ERCC3** ] , transactivation directed by certain nuclear receptors is inhibited, regardless of TTD versus XP phenotype, thus explaining the overlapping symptoms.  Ref: 12820975 Mol Cell, 2003
- General transcription factor IIH TFIIH  [ **GTF2H1** ]   [ **ERCC3** ]  consists of nine subunits cyclin dependent kinase 7 Cdk7, cyclin H and MAT1 forming the Cdk activating kinase or CAK complex, the two helicases Xpb  [ **ERCC3** ]  Hay and Xpd, and p34, p44, p52 and p62 refs 1 3.  Ref: 12853965 Nature, 2003

**Non-interactive relationship** (e.g. studied together, co-existance, homology, etc.)

- Here we show that the Drosophila TFIIH  [ **GTF2H1** ]   [ **ERCC3** ]  component Xpd negatively regulates the cell cycle function of Cdk7, the CAK activity.  Ref: 12853965 Nature, 2003
- As the kinase subunit of TFIIH  [ **GTF2H1** ]   [ **ERCC3** ] , Cdk7 participates in basal transcription by phosphorylating the carboxy terminal domain of the largest subunit of RNA polymerase II.  Ref: 12853965 Nature, 2003
- Together with recent reports linking TFIIH  [ **GTF2H1** ]   [ **ERCC3** ]  in regulating NR function, our findings provide new data to further support an important link between DNA repair proteins and nuclear receptor function.  Ref: 12874288 J Biol ChemJ Biol Chem, 2003
- The archaeal transcriptional machinery is polII like but does not require ATP or TFIIH  [ **GTF2H1** ]   [ **ERCC3** ]  for open complex formation.  Ref: 12783891 J Biol Chem< MedlineTA>J Biol Chem, 2003
- We also show that TFIIH  [ **GTF2H1** ]   [ **ERCC3** ]  from TTD patients, but not from XP patients, exhibits a significant in vitro basal transcription defect in addition to a reduced intracellular concentration.  Ref: 12820975 Mol Cell, 2003
- The preferred substrate for SCP1 is RNAP II phosphorylated by TFIIH  [ **GTF2H1** ]   [ **ERCC3** ] .  Ref: 12721286 J Biol Chem< MedlineTA>J Biol Chem, 2003
